# AI-Driven quality assurance in mammography: Enhancing quality control efficiency through automated phantom image evaluation in South Korea

**DOI:** 10.1371/journal.pone.0330091

**Published:** 2025-09-08

**Authors:** Hoo Yun, Sanghyun Noh, Hyungwook Cho, Eun Yong Ko, Zepa Yang, Ok Hee Woo

**Affiliations:** 1 Korea University College of Medicine, Seoul, Republic of Korea; 2 Hong Kong University of Science and Technology, Hong Kong; 3 Seoul National University College of Medicine, Seoul, Republic of Korea; 4 Department of Radiology, Samsung Medical Center, Seoul, Republic of Korea; 5 Department of Computer Engineering, Soonchunhyang University, Asan, Republic of Korea; 6 Department of Radiology, Korea University Guro Hospital, Seoul, Republic of Korea; King Abdulaziz University, SAUDI ARABIA

## Abstract

**Purpose:**

To develop and validate a deep learning-based model for automated evaluation of mammography phantom images, with the goal of improving inter-radiologist agreement and enhancing the efficiency of quality control within South Korea’s national accreditation system.

**Materials and methods:**

A total of 5,917 mammography phantom images were collected from the Korea Institute for Accreditation of Medical Imaging (KIAMI). After preprocessing, 5,813 images (98.2%) met quality standards and were divided into training, test, and evaluation datasets. Each image included 16 artificial lesions (fibers, specks, masses) scored by certified radiologists. Images were preprocessed, standardized, and divided into 16 subimages. An EfficientNetV2_L-based model, selected for its balance of accuracy and computational efficiency, was used to predict both lesion existence and scoring adequacy (score of 0.0, 0.5, 1.0). Model performance was evaluated using accuracy, F1-score, area under the curve (AUC), and explainable AI techniques.

**Results:**

The model achieved classification accuracy of 87.84%, 93.43%, and 86.63% for fibers (F1: 0.7292, 95% bootstrap CI: 0.711, 0.747), specks (F1: 0. 7702, 95% bootstrap CI: 0.750, 0.791), and masses (F1: 0.7594, 95% bootstrap CI: 0.736, 0.781), respectively. AUCs exceeded 0.97 for 0.0-score detection and above 0.94 for 0.5-score detection. Notably, the model demonstrated strong discriminative capability in 1.0-score detection across all lesion types. Model interpretation experiments confirmed adherence to guideline criteria: fiber scoring reflected the “longest visible segment” rule; speck detection showed score transitions at two and four visible points; and mass evaluation prioritized circularity but showed some size-related bias. Saliency maps confirmed alignment with guideline-defined lesion features while ignoring irrelevant artifacts.

**Conclusion:**

The proposed deep learning model accurately assessed mammography phantom images according to guideline criteria and achieved expert-level performance. By automating the evaluation process, the model can improve scoring consistency and significantly enhance the efficiency and scalability of quality control workflows.

## Introduction

Mammography plays a pivotal role in the early detection of breast cancer, and maintaining high-quality imaging standards is essential for accurate diagnosis. In South Korea, medical institutions operating mammography equipment are required to submit an annual quality control report, along with supporting documentation and a phantom image, to a government-authorized oversight body [1,2]. At this agency, the submitted phantom images are independently reviewed by two certified radiologists. In cases of disagreement, three additional panels review the image, and the results are determined by majority vote.

While this rigorous quality control protocol safeguards diagnostic quality, it imposes a considerable burden on radiologists who participate on panels, especially amidst the ongoing specialist shortage in South Korea [3]. The multilayered review process—particularly when additional expert panels are needed—can delay image approval and disrupt clinical workflows. These delays may also hinder timely breast cancer screening and diagnosis. Furthermore, discrepancies between institutional self-assessments and official reviews often trigger appeals, further complicating the administrative burden.

In recent years, artificial intelligence (AI) has shown great promise in advancing medical image interpretation, including mammography [4–13]. Deep learning models have demonstrated impressive capabilities in detecting and classifying abnormalities with performance levels comparable to those of expert radiologists [11,14]. While these advancements have largely been directed at diagnostic tasks, the same techniques also can be leveraged for quality control applications. In particular, mammography phantom image assessment—an inherently rule-based and repetitive task—represents a strong candidate for AI-driven automation [15]. By providing immediate, objective, and reproducible assessments, an AI-based system could significantly reduce evaluation time, improve scoring consistency, and enhance the scalability of quality assurance workflows.

Several studies have explored AI-based approaches to automating mammography phantom image evaluation [2,12,16,17]. Kretz et al. [16] (2019) trained a convolutional neural network (CNN) using real and simulated CDMAM phantom images. Sundell et al [12].(2022) proposed a CNN-based end-to-end pipeline that outputs binary scores for each of 17 classes in phantom images. Oh et al. [2] (2022) applied an interpretable YOLOv2 model across multiple institutions, enabling identification of ambiguous features. Park et al [10]. (2023) developed a filtering algorithm based on VGG16 that integrates multi-class and binary classifiers to reduce clinician workload. Although these studies solely rely on pure neural networks automate mammography quality assurance, there is room for improvement by implementing recent advancement in image detection that guides neural network to learn features with conventional methods [18–20].

However, few prior studies have explicitly focused on integrating explainable AI techniques with guideline-based scoring criteria to resolve ambiguity in borderline (score of 0.5) lesions — cases that critically influence final qualification decisions [11,21]. Building on these efforts, the present study aims to develop and validate a deep learning-based model for automated evaluation of mammography phantom image adequacy. Rather than merely accelerating review times, our goal is to demonstrate how AI can enhance consistency and transparency in quality control scoring—particularly in edge cases such as borderline lesions—while supporting broader standardization across healthcare systems. Ultimately, we propose automated AI as a robust foundation for scalable, high-quality mammography quality assurance.

## Materials and methods

### Data acquisition and preprocessing

Mammography phantom images submitted to the Korea Institute for Accreditation of Medical Imaging (KIAMI) by various medical institutions in Korea were collected for this study. The dataset includes images from several mammography phantom models recognized by the American College of Radiology (ACR), such as Z-710, T-540, and V-771, obtained using different mammography systems (film-based, computed radiography [CR], and digital radiography [DR]).

**Fig 1:**
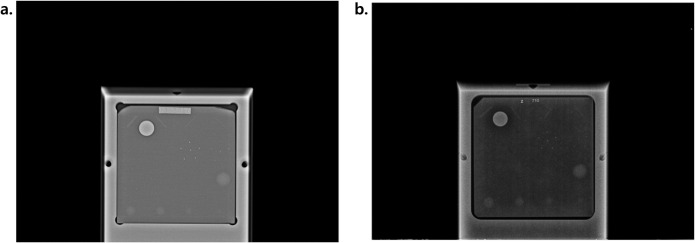
Sample phantom images. (a) Qualified – fiber: 6.0, specks: 4.0, mass: 4.0 (b) Unqualified – fiber: 1.5, specks: 4.0, mass: 1.5.

The mammography phantoms used for quality assurance testing contain 16 artificial lesions arranged in a 4 × 4 grid, categorized into three lesion types: fiber, specks, and mass. Each lesion is scored as 0.0, 0.5, or 1.0 depending on visibility and correct anatomical location [22,23]. Both the phantom images and their corresponding quality scores were obtained from KIAMI guidelines of which consider, a phantom image to have passed the quality control test if it demonstrates clear visualization of at least the four largest fibers, three largest speck groups, and three largest masses. Representative examples of both qualified and unqualified phantom images are shown in Fig 1.

All phantom images were reviewed by a panel of radiologists with more than 10 years of experience in mammography and certified membership in the Korean Society of Breast Imaging (KSBI) to ensure the reliability of the scoring system used in this study. For consistency and reproducibility, detailed scoring criteria were established for each lesion type in accordance with the KSBI quality assurance guidelines. Two breast imaging radiologists independently assessed each lesion, and if consensus was not achieved between the two readers, the case was escalated to an additional panel of three independent experts, and the final score was determined based on the majority opinion among the total of five radiologists. This multi-tiered adjudication process is part of the standardized national quality assurance program for mammography established in South Korea.

Scoring was based on lesion visibility and correct localization [24]. For fiber, a score of 1.0 was assigned only when the entire fiber was clearly visible without any breaks and correctly located and oriented. A score of 0.5 was given when more than half of the fiber was visible and properly aligned. For specks, a score of 1.0 was awarded if more than 4 of 6 specks were distinctly visible and correctly located, while 2–3 visible specks in the correct location received a score of 0.5. For mass, a score of 1.0 was given only if the lesion maintained a nearly circular shape—defined as greater than three-quarters of the contour appearing circular—and was located correctly. If the mass was not visible at the expected location, a score of 0.0 was then assigned.

A phantom image is considered qualified if the total scores for fibers, specks, and masses are at least 4.0, 3.0, and 3.0 respectively. Lesions that are smaller than the lowest-scoring lesion (i.e., lesions scoring 0.5 or 0.0) are ignored during qualification.

**Fig 2:**
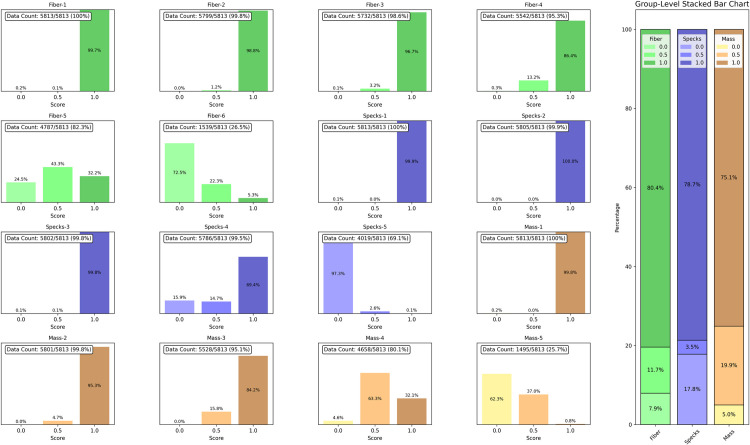
Grid-level and group-level score distribution of mammography phantom images.

A total of 5,813 phantom images was set as total dataset, of which 4,029 passed and 1,784 failed the quality assurance test. The dataset (n = 5,813) was split into training (70%), test (10%), and evaluation (20%) subsets which is a commonly adopted standard in deep learning-based medical imaging studies. Stratified sampling was applied to ensure proportional distribution of pass/fail outcomes across all subsets. (Table 1). The test set, derived from training, was used for model tuning and early stopping. The evaluation set was completely held out and used solely for final performance assessment. Each image was divided into 16 subimages. For model training, only lesions with a score of 1.0 and the largest lesion that failed to receive a score of 1.0 (those with 0.5 or 0.0) were included (Table 2). The same selection criteria were applied to the test set, while the evaluation set was used only for final performance measurement. A detailed visualization of the 16 individual lesions—fiber-1 to fiber-6, specks-1 to specks-5, and mass-1 to mass-5—and their group-level distributions is provided (Fig 2). To address limitations in the threshold-based scoring system, we introduced an additional score label, ‘N/A’, for ambiguous cases where a lesion might be visible but did not meet the visibility threshold for a score of 0.5. This addition reduced subjectivity and lowered the number of lesions classified as 0.0 or 0.5, particularly among higher-numbered lesions. This pattern is reflected in their reduced frequency within the dataset (Fig 2).

**Table 1:** Dataset split and qualification status for model training, test, and evaluation.

Dataset	Pass	Fail	Total
Training	2,820 (48.5%)	1,249 (21.5%)	4,069 (70.0%)
Test	403 (6.9%)	178 (3.1%)	581 (10.0%)
Evaluation	806 (13.9%)	357 (6.1%)	1,163 (20.0%)
Total	4,029 (69.3%)	1,784 (30.7%)	5,813 (100.0%)

**Table 2:** Distribution of lesion scores by feature type across training, validation, and evaluation datasets.

Dataset		Fibers	Specks	Mass
Training	0.0	1,568 (7.8%)	3,333 (17.8%)	802 (5.0%)
	0.5	2,365 (11.8%)	647 (3.5%)	3,175 (19.9%)
	1.0	16,068 (80.3%)	14,698 (78.7%)	12,008 (75.1%)
	Total	20,001	18,679	15,985
	Exclude	3,914	1,250	3,944
Test	0.0	261 (7.8%)	556 (17.9%)	134 (5.0%)
	0.5	394 (11.8%)	108 (3.5%)	529 (19.9%)
	1.0	2,678 (80.3%)	2,450 (78.7%)	2,001 (75.1%)
	Total	3,333	3,113	2,664
	Exclude	652	208	657
Evaluation	0.0	491 (8.4%)	957 (17.6%)	218 (4.7%)
	0.5	652 (11.1%)	205 (3.8%)	941 (20.3%)
	1.0	4,735 (80.6%)	4,271 (78.6%)	3,487 (75.1%)
	Total	5,878	5,433	4,646
	Exclude	1,094	377	1,164

### Image standardization and preprocessing

Preprocessing is critical for ensuring consistency, quality, and reproducibility in machine learning-based classification of mammography phantom images. Due to variations in scanner types, resolutions, and imaging conditions across institutions, a robust preprocessing pipeline was developed to normalize image properties and eliminate extraneous features.

**Fig 3:**
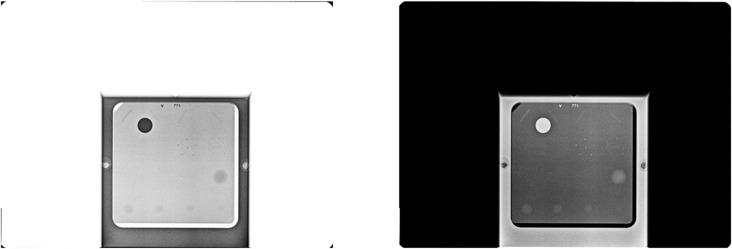
Conversion from monochrome1 to monochrome2 with background standardization.

1 Image format conversion:

DICOM images were converted to NumPy format for efficient handling of large datasets, as DICOM files often contain issues such as file corruption, unsupported tags, or missing metadata. Monochrome1 images were also inverted to Monochrome2 to ensure a consistent appearance (phantoms and lesions appear white against a black background), which improved interpretability (Fig 3).

**Fig 4:**
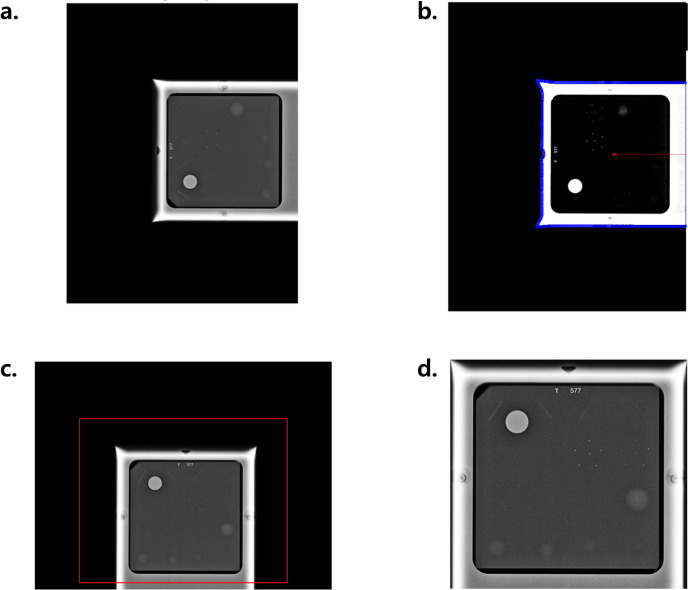
Phantom detection and alignment via thresholding and contour detection. (a) An original image. (b) An original image’s contour is detected with an Otsu method with threshold of 97 (blue). The center of mass of the region is obtained and its closest side is determined (red). (c) Rotation is applied to correct phantom orientation. (d) The image is cropped to the region of interest.

2 Phantom localization and alignment:

Images were rotated to place the phantom at the bottom center. Initial cropping removed irrelevant elements such as white borders, embedded text, and large black margins, to isolate the phantom. Phantom regions were detected using Otsu’s thresholding and OpenCV-based contour detection, and were fine-tuned to handle non-standard phantom placements (Fig 4).

**Fig 5:**
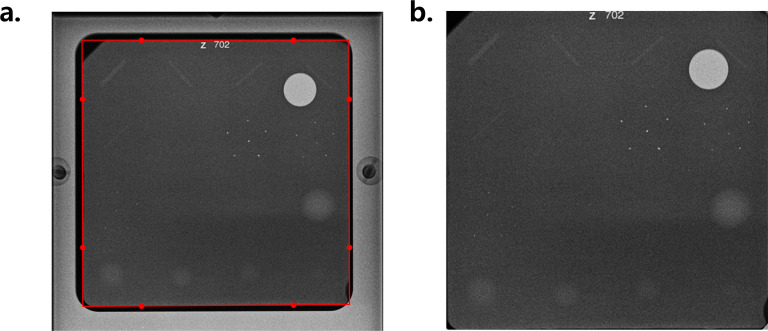
Lesion area isolation and precise cropping. (a) Only the region containing the gel is selected by analyzing the line profile into (b) the selected phantom image.

3 Lesion area cropping:

Using line-profile analysis on the initially cropped image, the border points of the lesion-containing region were identified to allow precise cropping. The resulting image was rotated slightly, if necessary, to ensure right-angle alignment of the lesion grid, standardizing the dataset for consistent analysis (Fig 5).

**Fig 6:**
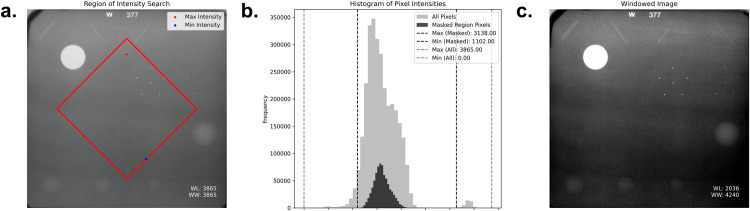
Normalization of pixel intensities and image standardization. (a) Pixel intensity of the rhombus was chosen for window adjustment to neglect any outlier such as the disk in the image. (b) The maximum and minimum value of the rhombus were chosen for normalization. (c) Normalized image.

4 Normalization and grid division:

Pixel intensities within the central region of each image were normalized to a 0–1 range to reduce the influence of extreme brightness or darkness (e.g., bright white circles or black backgrounds), while preserving local contrast essential for lesion detection. Normalization was performed prior to cropping to ensure consistent intensity across all lesions. All images were resized to 224 × 224 pixels and divided into 16 subimages using a uniform grid, with each subimage containing a single lesion (Fig 6).

**Fig 7:**
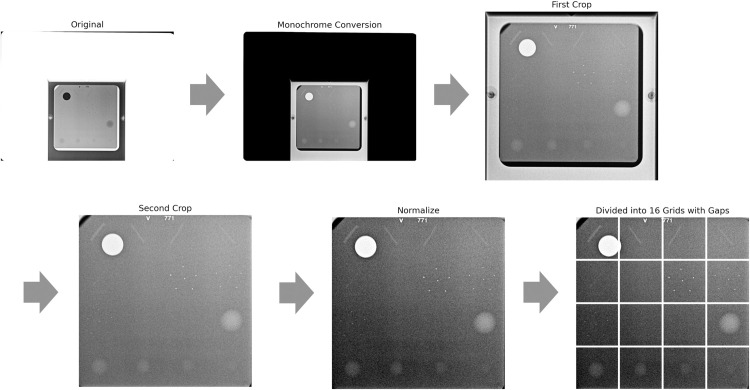
Visualization of the full workflow.

Of the 5,917 initially collected images, 5,813 (98.2%) were successfully processed using this structured preprocessing pipeline, which effectively transformed mammography phantom images into a consistent and standardized format (Fig 7). The remaining 104 images failed preprocessing due to issues such as excessive noise, flipped or severely rotated during acquisition, or non-standard phantom appearances with high variability that exceeded the algorithm capacity. These exclusions were made following a combination of rule-based filtering and visual inspection to ensure dataset integrity. While such cases were relatively infrequent, they reflected the variability inherent in real-world imaging environments. Nevertheless, the finalized dataset achieved a high level of quality and consistency, offering a robust foundation for downstream machine-learning applications.

### Model training

Image augmentation techniques were applied during model training to enhance generalization and reasoning capability. All lesion types were subjected to a combination of horizontal and vertical flipping, translational shifting, rotation, rescaling, and Gaussian blurring or noise addition.

The model architecture was based on EfficientNetV2_L, proposed by Tan and Le [25] (2021), and was designed to output two probabilities: one predicting the presence of a lesion and the other detecting abnormal features insufficient for a full-point classification [26,27]. For example, a 0.5-score label was encoded as [1, 1], with all other labels mapped accordingly.

The model was fine-tuned for 200 epochs using the mammography phantom dataset, starting from weights pretrained on the ImageNet-1k dataset. Stochastic gradient descent (SGD) was used as the optimizer, with a learning rate of 5e-3 for both the encoder and classifier layers and a weight decay of 1e-5 applied to each. To address class imbalance and assign greater importance to the 0.5-score category, focal loss with inverse proportion class weights with sigmoid activation function was adopted [28,29]. Loss of abnormality class was masked for 0.0-scored subimages when training [28,29].

### Model evaluation

The final model performance was evaluated using the evaluation dataset (20%), which remained completely isolated from both training and test stages. All reported metrics (accuracy, F1-score, AUC) were calculated based on this evaluation set. A 95% confidence interval for F1-score and AUC was estimated using bootstrapping with 10,000 resamples to quantify statistical reliability. The performance of the model was evaluated at multiple hierarchical levels. First, each subimage was independently scored, and the classification performance was assessed using accuracy and F1 score at the lesion level.

For feature-level quality assurance, an overall score for each feature (fiber, specks, and mass) was calculated by summing the individual subimage scores. To replicate the real-world evaluation process used in clinical settings, only the largest lesion that did not receive a score of 1.0 was considered when calculating the overall score for each feature [2,10]. According to the Korean quality assurance criteria, a feature was considered disqualified if its total score was less than 4.0 for fibers, 3.0 for specks, or 3.0 for masses. A phantom image was disqualified if any of its features were disqualified. Precision, recall, and accuracy were calculated for each feature as well as for the overall phantom-level classification.

While traditional metrics such as accuracy and F1-score offer useful insights, they are insufficient for fully capturing model behavior. To better interpret the model’s decision process, we generated image-specific class saliency maps based on the method proposed by Simonyan et al. [30] (2017). These maps approximate the neural network output using a first-order Taylor expansion and highlight the pixels that most strongly influence the model predictions [9,31].

## Results

### Performance evaluation of the mammography phantom image by the model

**Fig 8:**
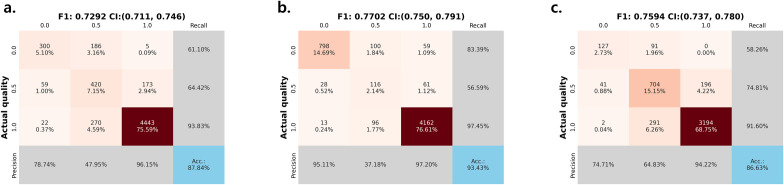
Confusion matrix of model predictions on the evaluation dataset for (a) fiber, (b) specks, and (c) mass. Accuracy, precision, and recall values are shown for each score class.

The trained model demonstrated strong performance in multigroup classification of individual subimages in the evaluation dataset (Fig 8). The classification accuracy and F1-scores were 87.84% and 0.7292 (95% bootstrap CI: 0.711, 0.747) for fibers, 93.43% and 0.7702 (95% bootstrap CI: 0.750, 0.791) for specks, and 86.63% and 0.7594 (95% bootstrap CI: 0.736, 0.781) for masses, respectively. Among the score categories, 0.5-score lesions were the most difficult to classify, yielding recall rates of approximately 60% and precision below 50% for fibers and specks, and 65% for masses. These results suggest that the model performs reliably for clearly visible 0.0-score or 1.0-score lesions, while showing reduced discriminability in borderline 0.5-score lesions.

**Fig 9:**
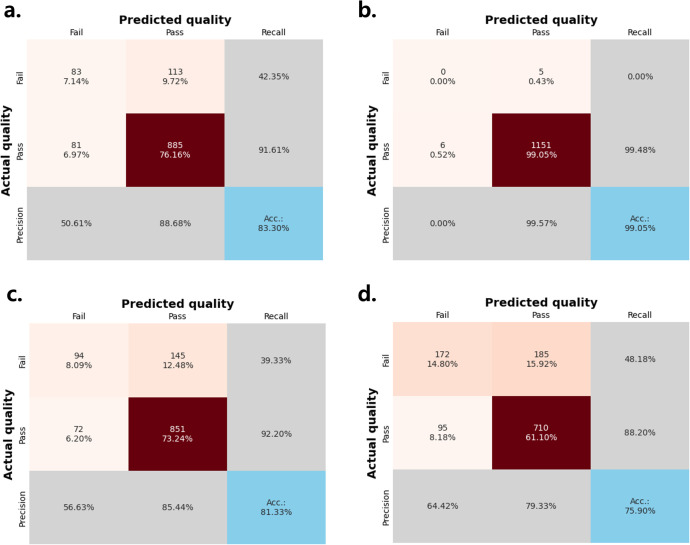
Receiver operating characteristic (ROC) curves and corresponding area under the curve (AUC) of the model prediction at the evaluation dataset for (a) fiber, (b) specks, and (c) mass. The existence of a lesion, and abnormality insufficient for a 1.0-score classification was presented as orange and green line, respectively. The threshold for each output is chosen to maximize the F1 score. Subimages with 0.0-score were excluded when plotting the abnormality ROC curve.

The model’s discriminative ability was further evaluated using receiver operating characteristic (ROC) analysis [32,33] (Fig 9), which incorporated its two probabilistic outputs for each sub-image—one estimating lesion presence and the other lesion abnormality. For the ROC curve representing lesion presence, sub-images assigned scores of 1.0 or 0.5 were treated as positive cases, whereas those scored 0.0 were considered negative. The abnormality ROC curve was constructed from the subset of sub-images scored as 1.0 or 0.5; within this subset, those labeled 0.5-score were designated positive for abnormality, and the 1.0-score cases served as negatives. This approach ensured that abnormality was evaluated solely in images where a lesion had been detected, as images without any lesion (score 0.0) provided no basis for abnormality assessment. The AUC values for lesion abnormality exceeded 0.94 for all features, with the highest values observed for specks. For lesion presence classification, all features yielded AUCs above 0.97. These findings demonstrate that the model exhibits high sensitivity and specificity across both lesion types and all score categories.

**Fig 10:**
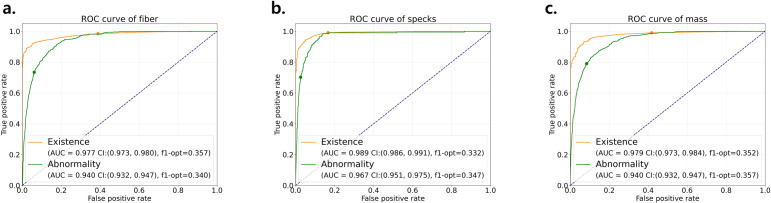
Confusion matrix of pass/fail predictions by feature. Each of the feature groups: (a) fiber, (b) specks, (c) mass, and (d) all three features are scored by the model, and validated as pass or fail. Only images that meet the requirements for all three features are considered qualified.

The utility of the model in binary classification for phantom image qualification was assessed by applying predictions to all 16 subimages per case and terminating the classification process upon detection of a lesion with a score of 0.5 or 0.0 (Fig 10). The feature-level classification accuracies were 83.30% for fibers, 99.05% for specks, and 81.33% for masses. However, relatively high false positive rates were noted, particularly in fibers and masses, as reflected in recall values of 42.35% and 48.18%, respectively. Despite these limitations, the model achieved an overall accuracy of 75.90% for phantom-level pass/fail determination.

### Analysis of the model logic in score predictions

**Fig 11:**
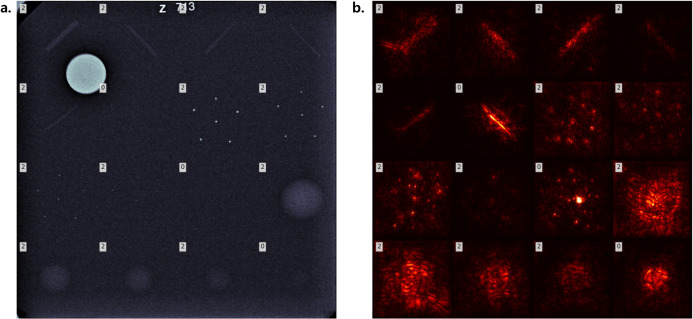
Visualization of (a) the phantom image and (b) corresponding saliency map. The annotations in the top-left corner of each subimage in (a) represent the labels while those in (b) represent the predictions, where 0, 1, and 2 correspond to scores of 0.0, 0.5, and 1.0, respectively. The saliency map is visualized via heatmap with the maximum value consistent across the same feature.

In the qualitative analysis, model attention was visualized through saliency maps for each subimage. An example from the evaluation dataset shows all 16 subimages reconstructed into a full phantom image along with their corresponding saliency maps, with subimage scores annotated in the upper-left corner of each tile (Fig 11). The heatmaps represent normalized saliency values, where the maximum was set to twice the 99.5% within each lesion type to ensure scale consistency.

**Fig 12:**
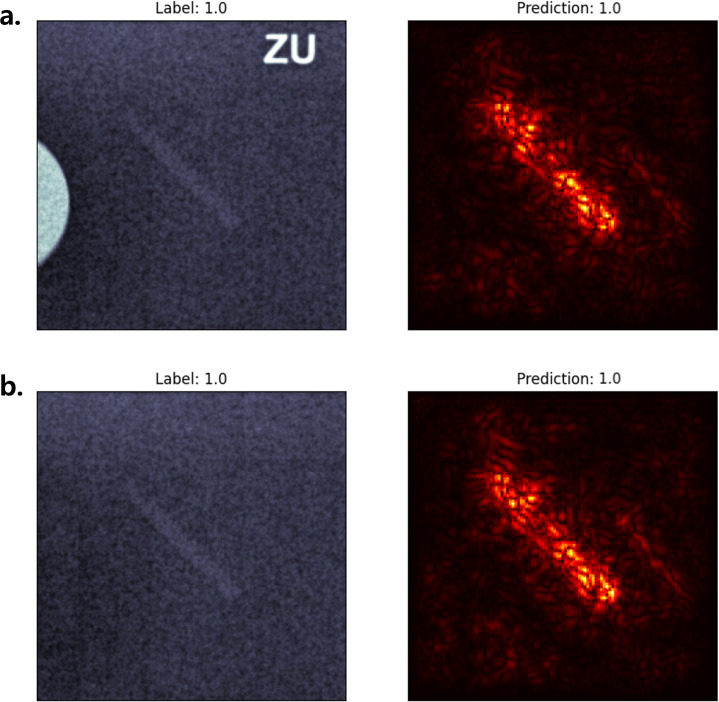
The model ignores artifacts and concentrates on the fiber itself. (a) A subimage of a fiber with artifacts such as letters and discs and (b) a masked subimage were scored by the model. The prediction and the saliency map were consistent before and after masking the artifacts, despite having been trained on images containing artifacts.

The model demonstrates robustness against visual artifacts embedded in phantom images. Predictions remained stable before and after masking non-lesion artifacts such as letters or discs, consistently focusing on the lesion regions rather than irrelevant elements (Fig 12). This suggests that the model attention is lesion-specific, despite being trained on images containing such artifacts.

**Fig 13:**
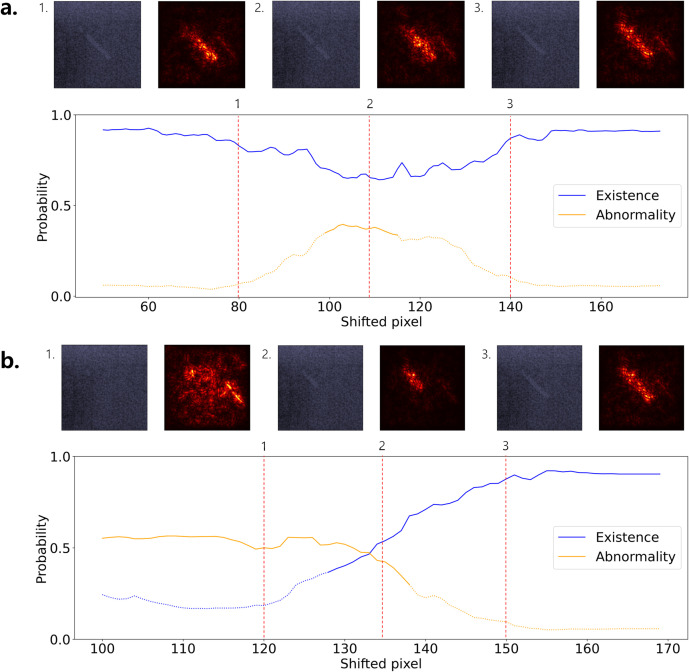
Model interpretation for fiber scoring based on visible length. (a) Saliency maps and class probabilities as the fiber is masked at different break positions; central breaks trigger the highest abnormality detection. (b) Saliency maps with corresponding class probabilities as fiber length increases; once a sufficient length is visible, the model assigns a score of 1.0 (no abnormality). Solid lines indicate predictions above the decision threshold, whereas dotted lines indicate predictions below it.

Model interpretation of fiber scoring was analyzed by varying two key attributes: the position of breaks along the fiber and the visible fiber length (Fig 13). The model produces two probabilities for each subimage: one estimating the presence of a lesion and the other detecting any abnormality insufficient for a score of 1.0. In visualizing these outputs, straight lines represent probabilities above the decision threshold, while dotted lines indicate probabilities below it. To examine the model sensitivity to break position, saliency maps and class probabilities were generated for subimages in which artificial breaks were introduced at different locations (Fig 13a). Breaks placed near the center of the fiber resulted in the highest abnormality probabilities, while peripheral breaks had a weaker effect. This pattern suggests that the model implicitly prioritizes the longest continuous fiber segment, consistent with clinical guidelines that emphasize fiber continuity in quality assurance scoring. The influence of fiber length was investigated by progressively unmasking portions of the fiber to increase its visible length (Fig 13b). As the fiber became longer, the model initially predicted its existence (Fig 13b-2); when the length surpassed a certain threshold, the model predicted normality. This corresponded to a score of 1.0 (Fig 13b-3). Notably, the probability for existence began to increase before any observed decrease in the abnormality probability, suggesting that the model views existence and abnormality as two distinct and independently assessed features. This two-phase response implies a reasoning structure in which the model first determines whether a lesion is present and only then evaluates its completeness or quality.

**Fig 14:**
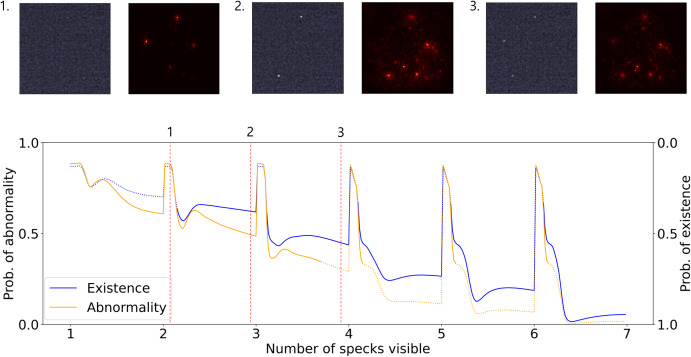
Model interpretation for speck scoring, with predictions grouped as “Existence” (lesion presence) and “Abnormality” (lesions insufficient for full-point qualification). Saliency maps and class probabilities are shown as the number of visible specks increases from 1 to 6 and speck visibility increases from 0 to 1. The model predicts 0.5 or 1.0-score at ≥2 visible specks, and 1.0-score at ≥3–4 specks. Solid lines indicate predictions above the decision threshold, whereas dotted lines indicate predictions below it.

The model scoring logic for specks was examined by manipulating the number and visibility of specks in a controlled manner (Fig 14). Beginning with a fully masked 1.0-score subimage, specks were incrementally unmasked with varying opacities to simulate partial visibility. When no specks were visible, the model consistently predicted a 0.0-score classification, indicating no lesion (Fig 14-1). As the number of specks increased beyond 2, the model began assigning score of 0.5, interpreting the subimage as containing a lesion with abnormal characteristics (Fig 14-2). Once the number of visible specks exceeded 3−4, the model reliably predicted a 1.0-score classification, indicating normal lesion appearance (Fig 14-3). Interestingly, the class probability curves exhibited distinct transition patterns depending on the speck count. When 1–3 specks were visible, the probability dropped once and then plateaued, whereas the curve for 4–6 specks showed a 2-phase decline before stabilization. This suggests that the model performs an internal thresholding process that aligns with the graded criteria used in human visual scoring, where both quantity and clarity of specks are key determinants.

**Fig 15:**
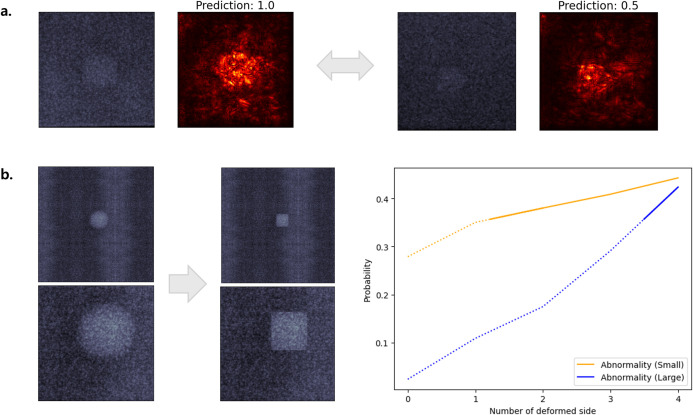
Model interpretation for mass scoring. (a) Saliency maps show correct shape localization but a size bias: larger lesions predicted as 1.0-score, smaller as 0.5-score despite identical label. (b) Probability of abnormality increases as the image is deformed into a square shape. Both sizes show increasing abnormality with deformation, with the smaller lesion consistently yielding higher probabilities. Final probabilities converge once deformation is complete. Solid lines indicate predictions above the decision threshold, whereas dotted lines indicate predictions below it.

The model’s ability to detect shape abnormalities in masses and its sensitivity to lesion size were investigated using progressive shape deformation and rescaling experiments (Fig 15). Two subimages with similarly square-shaped lesions—both labeled as 0.5 due to shape irregularity—were compared. Despite equivalent deformation, the model predicted a score of 1.0 for the larger lesion and a score of 0.5 for the smaller one, indicating a size-dependent bias (Fig 15a). Further analysis involved gradual masking of a circular mass (originally scored as 1.0) into a square shape while tracking the predicted probability of abnormality. As deformation increased, the abnormality score also increased, confirming the model sensitivity to loss of circularity (Fig 15b). When the same image was rescaled to one-third of its original size, the abnormality probability increased more rapidly than in the original image. However, upon square deformation, both the small and large lesions converged to a similar abnormality probability. These findings suggest that, while the model accurately detects shape abnormalities, its predictions are modulated by lesion size.

## Discussion

A key insight from this study is the disproportionate influence of 0.5-score lesions on the qualification of mammography phantom images.. The phantom scoring system established by the Korean Imaging Evaluation Center intentionally limits the frequency of ambiguous 0.5-score lesions. While appropriate for human evaluation, this presents intrinsic challenges for data-driven AI training. Even when the overall lesion visibility appears acceptable, subtle abnormalities—such as minor fiber discontinuities or slightly irregular mass shapes—frequently lead to 0.5 scores. These borderline cases can lead to disqualification of the entire phantom image, especially when abnormalities in larger lesions cause smaller lesions to receive a score of 0.0. Although various training strategies were attempted to enhance performance in this class, consistent gains were not achieved. Future research with larger dataset or synthetic generation methods would lead to better represent borderline lesions.

While the proposed model performed well in detecting 1.0-score lesions, achieving AUC values larger than 0.95 across each model classes, its performance in scoring 0.5-score lesions revealed persistent ambiguity. The model tended to over-predict abnormality in these cases, as reflected by skewed ROC curves, indicating a conservative decision-making pattern. The observed false-positive rates in fiber and mass features likely reflect inherent variability in image interpretation and lesion visibility under expert consensus scoring. Similar results have been reported in previous studies (e.g., Park et al., 2023). Although such conservatism may be appropriate in safety-focused quality assurance settings, further calibration such as feature-specific calibration and rule-based ensemble techniques may be necessary to reduce false positives without compromising robustness. These findings underscore the importance of AI models that are not only highly accurate but also reliably interpret edge cases, acknowledging the inherent subjectivity and inter-observer variability.

In the binary pass/fail classification task, the model demonstrated strong performance in detecting non-qualifying phantom images. However, feature-wise precision varied significantly. While speck detection exhibited high accuracy, the classification of fibers and masses was less reliable, likely due to class imbalance and more complex morphological criteria [34]. Since phantom disqualification can result from failure in any single feature, misclassifications in more challenging categories like fiber can disproportionately influence the final evaluation. This highlights the importance of feature-aware thresholding strategies and confidence-calibrated ensemble outputs.

The model’s strong binary pass/fail performance at the phantom level belies important nuances in how lesion‐level errors propagate: because the overall QA decision hinges on per‐feature probability thresholds, using the multiclass F1‐optimal threshold—while not mathematically ideal for maximizing phantom‐level accuracy—supports human–machine collaboration by flagging ambiguous cases for review. Under this threshold, the system yields a higher false‐negative rate than false‐positive rate, which is advantageous in a screening context where the bulk of images are of sufficient quality and only truly problematic phantoms should interrupt workflow. This approach balances the need for efficient throughput of qualified images with the imperative to catch the relatively rarer but clinically critical quality failures.

Saliency map analyses further revealed that the model attention aligned well with human-interpretable lesion characteristics, such as fiber continuity, speck visibility, and mass circularity. The model largely ignored non-informative image artifacts like embedded text and background structures, even without explicit artifact masking, demonstrating robust focus on relevant features. This behavior supports the clinical utility of explainable AI (XAI) tools not only in model validation but also in building clinician trust [6]. Nonetheless, observed reliance on lesion size—particularly in mass evaluation—indicates a potential bias that could limit generalizability across imaging systems [35]. Incorporating size-normalized features or lesion-type-specific scaling strategies may improve model performance.

Model scoring exhibited dependence on lesion size, particularly in the evaluation of masses. Preliminary tests confirmed variation in pass–fail thresholds across different size variants, reflecting a size bias that partially aligns with expert visual assessment practices. However, other confounding factors—including noise patterns, contrast resolution, and imaging hardware characteristics—also contributed to score variability. Given the complexity of these interactions, a comprehensive analysis of size-normalized calibration was deemed beyond the scope of this study but will be undertaken in follow-up work to support consistent national QA implementation.

Beyond improving individual classification accuracy, this study suggests broader applications for AI in standardizing quality assurance practices. Variability in human scoring—especially in borderline cases—limits reproducibility across institutions. By providing consistent, transparent, and guideline-concordant evaluations, AI-based systems can contribute to scalable and objective quality assurance frameworks, both nationally and internationally. This potential is particularly relevant as imaging volumes increase and accreditation requirements become more stringent. For example, such systems could support centralized quality control efforts in national programs like those administered by KIAMI or promote inter-institutional consistency across multicenter hospital networks.

While this study focused on mammography phantom images submitted under South Korea’s national quality assurance standards (KIAMI), the evaluation was based on ACR-approved phantom models that are widely used internationally. Therefore, the core scoring principles are largely consistent across countries. However, specific components—such as the inclusion of addendum phantoms or magnetic adhesive parts—may vary by national protocols. For broader international deployment, additional model adaptation and fine-tuning may be necessary to meet country-specific scoring guidelines and hardware configurations.

To enhance the clinical utility of such models, several improvements should be considered. Ensemble modeling and calibration layers may help reduce misclassification between closely related categories such as 0.5 and 1.0-score lesions. Training with expert consensus labels or cross-reviewed datasets may also reduce ambiguity in 0.5-score lesions. Adaptive thresholds based on lesion type or size could improve classification granularity, while user-feedback mechanisms or hybrid rule-based overlays may support safer real-world deployment. The automation of the surveillance process may also enable real-time feedback when integrated into mammography systems, reducing the need for time-intensive centralized review.

This study has several limitations. First, the model was trained on image sets reviewed by different radiologists, limiting its capacity to disambiguate borderline cases. This study has several limitations. First, the model was trained on image sets reviewed by multiple radiologists, which limits its ability to disambiguate borderline cases. This limitation reflects the inherent nature of the QA procedure, since the current Korean national QA review process involves up to five board-certified radiologists to ensure high-confidence consensus scores [24,36].Inter-observer variability was mitigated through a multi-stage adjudication process built into the QA framework. Future studies comparing inter-observer variability with annotator–model agreement can quantitatively assess the model’s performance.

Second, the inherently subjective nature of mammography quality assurance complicates automated evaluation. Providing continuous scoring outputs or probabilistic confidence levels may offer better alignment with clinical judgment.

Third, while this study focused on evaluating ACR phantom images under the current regulatory framework, it did not explore stratified performance trends across imaging hardware or acquisition protocols. Future research will be required to assess generalizability to non-ACR phantom types and to validate robustness under varied clinical conditions. This pilot validation study is intended to serve as a foundational benchmark for such expanded investigations.

Last, trend-based metrics such as ROC curves may better reflect performance than discrete measures like accuracy. Developing a threshold-adjustable, computer-aided quality assurance framework would help address these challenges.

## Conclusion

The proposed deep learning model accurately assessed mammography phantom images according to guideline criteria and achieved expert-level performance and reduced inter-reader variability, especially in borderline lesions. By automating the evaluation process, the model can improve scoring consistency and significantly enhance the efficiency and scalability of quality control workflows. This approach offers a practical and reproducible solution for nationwide mammography QA systems. Future research should focus on improving the model precision in detecting borderline lesions and validating its generalizability across diverse clinical settings, equipment vendors, image formats, and phantom models.

## Supporting information

S1 Data Minimal data Figs 8 and 9 matrix. (CSV)

S2 Data Minimal data Fig 10 matrix. (CSV)

S3 Data Minimal data Fig 13 fiber. (CSV)

S4 Data Minimal data Fig 13 fiber 2. (CSV)

S5 Data Minimal data Fig 14 specks. (CSV)

S6 Data Minimal data Fig 15 mass. (CSV)

S1 File Sample phantom images (100 de-identified cases, part 1). (ZIP.001)

S2 File Sample phantom images (100 de-identified cases, part 2). (ZIP.002)

S3 File Sample phantom images (100 de-identified cases, part 3). (ZIP.003)

S4 File Sample phantom images (100 de-identified cases, part 4). (ZIP.004)

S5 File Label data for 100 sample phantom cases (fiber, speck, mass scores). (CSV)
